# Empirical antibiotic treatment for community-acquired pneumonia and accuracy for *Legionella pneumophila, Mycoplasma pneumoniae,* and *Clamydophila pneumoniae:* a descriptive cross-sectional study of adult patients in the emergency department

**DOI:** 10.1186/s12879-023-08565-6

**Published:** 2023-09-05

**Authors:** Morten Hjarnø Lorentzen, Flemming Schønning Rosenvinge, Annmarie Touborg Lassen, Ole Graumann, Christian B. Laursen, Christian Backer Mogensen, Helene Skjøt-Arkil

**Affiliations:** 1 Emergency Department, Hospital Sønderjylland, Aabenraa, Denmark; 2 Department of Regional Health Research, University of Southern Denmark, Odense, Denmark; 3 Clinical Microbiology, Odense University Hospital, Odense, Denmark; 4 Emergency Department, Odense University Hospital, Odense, Denmark; 5 Department of Clinical Research, University of Southern Denmark, Odense, Denmark; 6 Department of Radiology, Aarhus University Hospital, Aarhus, Denmark; 7 Department of Clinical Medicine, Aarhus University, Aarhus, Denmark; 8 Department of Respiratory Medicine, Odense University Hospital, Odense, Denmark; 9 Odense Respiratory Research Unit (ODIN), Department of Clinical Research, University of Southern Denmark, Odense, Denmark

**Keywords:** Pneumonia, CAP, Empirical Antibiotic treatment, Legionella, Infection

## Abstract

**Background:**

Many factors determine empirical antibiotic treatment of community-acquired pneumonia (CAP). We aimed to describe the empirical antibiotic treatment CAP patients with an acute hospital visit and to determine if the current treatment algorithm provided specific and sufficient coverage against *Legionella pneumophila, Mycoplasma pneumoniae,* and *Clamydophila pneumoniae* (LMC).

**Methods:**

A descriptive cross-sectional, multicenter study of all adults with an acute hospital visit in the Region of Southern Denmark between January 2016 and March 2018 was performed. Using medical records, we retrospectively identified the empirical antibiotic treatment and the microbiological etiology for CAP patients. CAP patients who were prescribed antibiotics within 24 h of admission and with an identified bacterial pathogen were included. The prescribed empirical antibiotic treatment and its ability to provide specific and sufficient coverage against LMC pneumonia were determined.

**Results:**

Of the 19,133 patients diagnosed with CAP, 1590 (8.3%) patients were included in this study. Piperacillin-tazobactam and Beta-lactamase sensitive penicillins were the most commonly prescribed empirical treatments, 515 (32%) and 388 (24%), respectively. Our analysis showed that 42 (37%, 95% CI: 28–47%) of 113 patients with LMC pneumonia were prescribed antibiotics with LMC coverage, and 42 (12%, 95% CI: 8–15%) of 364 patients prescribed antibiotics with LMC coverage had LMC pneumonia.

**Conclusion:**

Piperacillin-tazobactam, a broad-spectrum antibiotic recommended for uncertain infectious focus, was the most frequent CAP treatment and prescribed to every third patient. In addition, the current empirical antibiotic treatment accuracy was low for LMC pneumonia. Therefore, future research should focus on faster diagnostic tools for identifying the infection focus and precise microbiological testing.

**Supplementary Information:**

The online version contains supplementary material available at 10.1186/s12879-023-08565-6.

## Background

Community-acquired pneumonia (CAP) is one of the most common infections among patients in the emergency department and carries high mortality worldwide [1, 2]. Globally, the yearly incidence of CAP in adults is estimated to be between 1.5 and 14.0 per 1000 people, with short-term mortality for hospitalized patients between 4 and 18% [3].

The most frequent CAP pathogens, *Streptococcus pneumoniae* and *Haemophilus influenzae,* typically respond to narrow-spectrum beta-lactam antibiotics like benzylpenicillin in countries with low penicillin resistance [4, 5]. Other CAP pathogens like *Legionella pneumophila, Mycoplasma pneumoniae,* and *Clamydophila pneumoniae* (LMC) are uncommon, seldom cause severe pneumonia and can be treated with macrolides or quinolones [5, 6].

Indiscriminate use of antibiotics like macrolides and quinolones is problematic. Firstly, they are drivers of the increasing worldwide problem of antimicrobial resistance (AMR) [7, 8]. WHO has classified AMR as a major global threat, and antibiotics like third generation cephalosporins, macrolides, fluoroquinolones, piperacillin/tazobactam, and amoxicillin/clavulanic acid as critical antibiotics for humans, so unnecessary use should be limited to avoid resistance. Secondly, these antibiotics are associated with serious side effects, e.g. *Clostridium difficile* infection, cardiotoxicity and teratogenicity [9–12].

The microbiological etiology is rarely known when a patient is treated for suspected CAP, and empirical antibiotic treatment should be adjusted to local epidemiology and AMR patterns [2]. Guideline recommendations can be based on various severity scores such as the Pneumonia severity index (PSI), CRB-65, and CURB-65 [13]. Great Britain, Germany, and Denmark use the CRB-65/CURB-65 severity score approach to guide empirical antibiotic treatment for CAP [13–16]. These guidelines recommend penicillin, such as benzylpenicillin and amoxicillin, for patients with low CURB-65 scores. For patients with high CURB-65 scores, guidelines recommend coverage against LMC pathogens as a precaution. However, LMC pathogens are less common in patients with high CURB-65 scores [17, 18].

In addition to the guidelines, factors such as comorbidities, previous antibiotic treatment, community outbreaks of specific pathogens, treatment sites, antibiotic supplies, and the treating physicians’ knowledge and compliance with guidelines all impact the choice of empirical antibiotic treatment [19–21]. Despite the many factors determining the empirical antibiotic treatment of CAP and the increasing antibiotic resistance worldwide, we found little knowledge about which antibiotics are prescribed for patients with CAP and if the current guidelines based on scoring systems for pneumonia are successful in recommending antibiotic treatments that target the actual bacterial etiology.

Therefore, our aim was 1) to describe the type of empirical antibiotic treatment prescribed to CAP patients on arrival and 2) to determine if the current treatment algorithm provided specific and sufficient coverage against LMC pneumonia.

## Method

### Study design

This study was conducted in the Region of Southern Denmark, the third largest region in Denmark, with a population of 1.2 million. The region has four hospital units: Odense University Hospital, Lillebaelt Hospital, Hospital South West Jutland, and Hospital Sønderjylland.

We conducted a descriptive cross-sectional study based on the Southern Denmark Antibiotic Stewardship (SODAS) database. The database was established to evaluate the impact of the implementation of the Region of Southern Denmark’s antibiotic stewardship (RSDAS) in 2017 on individual antibiotic treatment [22]. SODAS consists of retrospective data collection from eight departments distributed across 4 hospitals receiving acute patients over 18 years. All data were extracted from the patient’s electronic medical record and supplemented with data from the Danish National Patient Registry [23].

RSDAS was implemented through campaigns and mandatory educational training [24, 25]. In addition, a comprehensive and rigorous material sampling for microbiological analysis was recommended.

Our study adheres to the STROBE guidelines for reporting observational studies in epidemiology [26].

### Participants

All patients from SODAS with a discharge diagnosis of pneumonia between January 2016 to March 2018 (International classification of diseases 10^th^ edition (ICD-10): DJ100, DJ111, DJ158, DJ159, DJ180, DJ189) were eligible (appendix 1). Patients with previous admissions within the last 14 days were excluded as they were deemed readmissions or hospital-acquired pneumonia. In addition, patients not receiving antibiotic treatment within the first 24 h of admission were excluded to ensure that only community-acquired infection was included. Only patients with identified bacterial infections were included in this study.

### Variables

Charlson Comorbidity Index (CCI) was calculated from the last ten years' discharge diagnoses prior to admission and grouped into three groups; 0, 1, ≥ 2 points [27]. CURB-65 score was not possible to calculate because the data contained no urea data, but CRB-65 was calculated when possible.

#### Antibiotic treatment

The last prescribed antibiotic treatment within 24 h of admission was regarded as the empirical treatment. Antibiotic guidelines for CAP are listed in Table 1. Before 2017 guidelines for antibiotic treatments for pneumonia were described in leading national textbooks [28]. In 2017 national and regional guidelines (RSDAS) were instituted.

**Table 1:** Empirical antibiotic guidelines for CAP before RSDAS (before 2017) and after RSDAS (2017 and beyond). Antibiotics are stated according to antibiotic class [22, 28]

	**Antibiotic guidelines before RSDAS**	**RSDAS antibiotic guidelines**
CURB-65 score: 0–2	- A beta-lactamase-sensitive penicillin OR - A macrolide* - Penicillin allergy: a macrolide	- A beta-lactamase-sensitive penicillin - Penicillin allergy: a macrolide or Cefuroxime
CURB-65 score: 3–5	- A beta-lactamase-sensitive penicillin + Fluoroquinolone - Penicillin allergy: Fluoroquinolone	- A beta-lactamase-sensitive penicillin + macrolide OR - Piperacillin/tazobactam + macrolide ** - Penicillin allergy: Cefuroxim + macrolide
Chronic obstructive pulmonary disease (COPD) exacerbations	- Amoxicillin/clavulanate	- Amoxicillin/clavulanate OR - Piperacillin/tazobactam*** - Allergy: Cefuroxime
Infection with unknown focus	- Piperacillin/tazobactam	- Piperacillin/tazobactam - Allergy: Meropenem

*Suspected Mycoplasma pneumoniae pneumonia, based on patient age or doing Mycoplasma pneumoniae outbreaks

**CURB-65 score ≥ 3 and hypoxia (SAT < 92%) or involving ≥ 2 lung lobes radiologically or sepsis

***Need for mechanical ventilation or oral treatment not possible

Information on penicillin allergy was not available.

For our second aim, prescribed macrolide or fluoroquinolone was assumed to reflect the application of treatment guidelines to ensure antibiotic coverage against LMC pneumonia. Tetracyclines are not recommended in Danish guidelines and are rarely used for CAP in Denmark.

#### Microbiology

The microbiological identified etiology of CAP was determined from standard clinical, microbiological testing. Guidelines for microbiological testing are described in appendix 2. A list of creditable CAP pathogens identified in sputum and blood culture was determined by a microbiologist (FSR) and an infectious disease consultant (CBM) (appendix 3). In cases with more than one identified pathogen, LMC pathogens were used if one was identified.

More extensive microbiological testing, Polymerase chain reaction (PCR) for LMC pathogens and treatment targeting LMC pathogens, were recommended for patients with CURB-65 score > 2, and a source of potential detection bias.

### Analysis

Patients' characteristics were reported with mean or proportion when relevant. Empirical antibiotic treatment and microbiological etiology were reported as quantity and percentage of the total. Empirical antibiotic treatment accuracy for LMC pneumonia was expressed as 1) quantity and proportion of LMC pneumonia prescribed antibiotic covering LMC pneumonia. 2) Quantity and proportion of patients prescribed antibiotics covering LMC with LMC pneumonia. 3) Number of prescribed antibiotics covering LMC pneumonia needed to correctly treat one LMC pneumonia. 95% confidence interval was calculated for proportions when appropriate.

Cases with missing microbiological etiology or empirical antibiotic treatment were excluded from the main analysis.

The sample size was determined by the number of cases in the SODAS cohort. Data analysis was done using STATA 17 (StataCorp, USA).

#### Ethics

The study was performed in accordance with the Helsinki declaration. According to the Danish Act on scientific ethical treatment of health science research projects, register based studies do not require ethics committee approval [29]. The need for informed patient consent was waived by the Danish Patient Safety Authority (record 3–3013-2272/1/), and the processing of personal data was approved by the Region of Southern Denmark and listed in the internal record (2012–58-0018 no. 17/24904) cf. art 30 of The EU General Data Protection Regulation.

## Results

### Participants

**Fig. 1:**
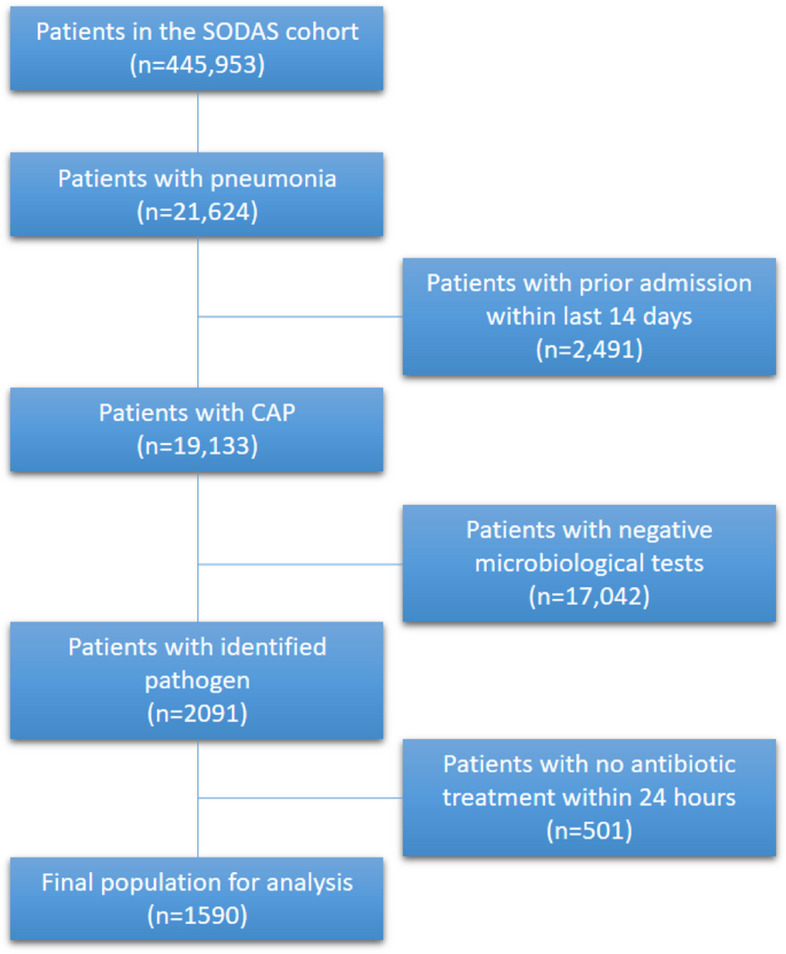
Flowchart of inclusion and exclusion

During the study period, a total of 443,953 contacts were included in the SODAS database, of which 21,624 had a discharge diagnosis of pneumonia (Fig. 1), 2,491 were excluded as readmissions within 14 days, resulting in 19,133 contacts for inclusion. We identified pathogen causes in 2,091 patients, of which 501 did not receive antibiotics within 24 h. In total, 1590 (8.3%) CAP patients were included in our analysis.

### Descriptive data

#### Patient characteristics

Characteristics of the study population are listed in Table 2. An extended list of antibiotics can be found in appendix 4. Identified viral pneumonias, the frequency of microbiological testing and the frequency of positive test can be found in appendix 5 and appendix 6.

**Table 2:** Patients characteristics, empirical antibiotic treatment and etiology for CAP in the study population

	**Total (*n* = 1590)**
**Male (%)**	831 (52%)
**Mean age in years (SD)**	70.0 (15.7)
**Comorbidities**	
- Chronic obstructive pulmonary disease (COPD) (%)	631 (40%)
- Congestive heart failure (%)	165 (11%)
- Diabetes without complications (%)	257 (16%)
- Diabetes with complications (%)	81 (5%)
- Median Charlson comorbidity score (IQR)	1 (0–2)
**Treating hospital and city of location (%)**	
- Hospital Sønderjylland	208 (13%)
- Hospital South West Jutland	157 (10%)
- Lillebaelt Hospital	391 (25%)
- Odense University Hospital	834 (52%)
**Empirical antibiotic treatment < 24 h after admission**	
- Piperacillin-tazobactam	515 (32%)
- Beta-lactamase-sensitive penicillins monotherapy	388 (24%)
- Combination therapy including macrolide	166 (10%)
- Cephalosporin	148 (9%)
- Fluoroquinolone monotherapy	113 (7%)
- Amoxicillin/clavulanate	96 (6%)
- Combination therapy including fluoroquinolone	38 (2%)
- Macrolide monotherapy	36 (2%)
- Amoxicillin or ampicillin	33 (2%)
- Antibiotics targeting urinary tract infections	13 (1%)
- Other	44 (3%)
***Etiology***	
- *Streptococcus pneumoniae*	396 (25%)
- *Haemophilus influenzae*	381 (24%)
- *Staphylococcus aureus*	315 (20%)
- *Pseudomonas aeruginosa*	176 (11%)
- *Moraxella catarrhalis*	128 (8%)
- *Hemolytic streptococci*	81 (5%)
- LMC-pneumonia	114 (7%)
*- Mycoplasma pneumonia*	79 (5%)
*- Legionella Pneumophila*	26 (2%)
*- Chlamydophila Pneumoniae*	8 (1%)
**CRB-65 score**	**(*n* = 872)**
- CRB-65 = 0	29 (3%)
- CRB-65 = 1	212 (24%)
- CRB-65 = 2	481 (55%)
- CRB-65 = 3	149 (17%)
- CRB-65 = 4	1 (< 0%)

### Main results

#### Antibiotic treatment

Monotherapy piperacillin-tazobactam and Beta-lactamase sensitive penicillins accounted for more than half of all empirical antibiotic treatments, with 515 (32%) and 388 (24%), respectively (Table 2).

#### Prescribed antibiotics coverage against LMC-pneumonia

Antibiotics with LMC coverage were prescribed to 364 (23%) totally, among these to 42 (37%, 95% CI: 28–47) of 113 LMC pneumonia patients and 42 (12%; 95% CI: 8–15) with prescribed LMC antibiotics had LMC pneumonia (Table 3). The number of patients prescribed LMC covering antibiotics needed to treat one LMC pneumonia was 8.7.

**Table 3:** Prescribed antibiotic coverages against LMC-pneumonia for LMC-pneumonia and non-LMC-pneumonia

	**LMC pneumonia**	**Non-LMC-pneumonia**	**Total**
Prescribed antibiotics covering LMC pneumonia	42	322	364
Prescribed antibiotics not covering LMC pneumonia	71	1155	1226
Total	113	1477	1590

### Discussion

Our study found that piperacillin-tazobactam and Beta-lactamase sensitive penicillins were the most prescribed empirical treatments, with 515 (32%) and 388 (24%), respectively. Additionally, our analysis showed that only 42 (37%, 95% CI: 28–47%) of patients with LMC pneumonia were covered by the empirical antibiotic treatment, while only 71 (12%, 95% CI: 8–15%) of patients treated with antibiotics with LMC coverage actually had LMC pneumonia.

#### Prescribed empirical antibiotics

The majority, 515 (32%) of all antibiotics prescribed were monotherapy Piperacillin-tazobactam. This was notwithstanding a shortage of Piperacillin-tazobactam from May to September 2017 [30]. Piperacillin-tazobactam monotherapy is the recommended empirical treatment for patients with an unknown infection focus, sepsis, or Chronic obstructive pulmonary disease (COPD) exacerbation requiring mechanical ventilation or where oral treatment were impossible [22]. Although some of the Piperacillin-tazobactam prescribed may have been for patients with COPD, only 96 (6%) of included patients were prescribed the first-line antibiotic, Amoxicillin/clavulanate. Suggesting that some COPD patients were prescribed piperacillin-tazobactam, instead of the recommended Amoxicillin/clavulanate. Additionally, the frequent use of monotherapy piperacillin-tazobactam and antibiotics targeting urinary tract infections indicate that the focus of infection is often uncertain in CAP patients at admission. Furthermore, it highlights a need for better diagnostic tools to determine the focus and microbiological etiology of the infection. Diagnostic uncertainty has previously been mentioned as a cause for the increased use of broad-spectrum antibiotics [31].

In a Danish study, 45% of CAP patients were treated with Beta-lactamase sensitive penicillins, compared to only 24% in our study [32]. The higher prescription rate of narrow-spectrum antibiotics may result from increased diagnostic certainty as the study used new infiltrates on chest x-ray as a criteria for diagnosing pneumonia which we did not. Although chest x-ray is the current first-line imaging tool for pneumonia, it has low sensitivity for pneumonia [33, 34]. Including only patients with new infiltration on chest x-ray excludes a significant number of CAP patients. In the future, tools like thoracic ultrasound and ultra-low-dose CT might improve focal diagnostics and facilitate focal targeted antibiotic treatment [35–37].

#### Prescribed antibiotic coverage against LMC pneumonia

We found that the empirical treatment guided by severity scores covering LMC pneumonia to be non-specific and insufficient. This result is not surprising, as studies assessing the ability of severity scores to predict the etiology of CAP reported a decreased frequency of LMC pneumonia in patients with high severity scores [18, 38]. In addition, other studies have shown that up to half of patients with low CURB-65 scores, where beta-lactam antibiotics were recommended, were treated with both a beta-lactam and a macrolide antibiotic [19–21]. Since empirical antibiotic coverage against LMC is based on a patient’s severity score, LMC pneumonia not treated with empirical antibiotics is likely to be less severe.

All these factors may explain our results: the prescription of antibiotics with LMC pneumonia coverage in 23% of patients despite LMC only being identified in 7% of patients, only 37% of patients with LMC pneumonia were prescribed antibiotics with LMC coverage, and only 12% (95% CI: 8–15) of patients prescribed antibiotic covering LMC had an LMC-pneumonia. This indicates a considerable potential for improvement in use of antibiotics with more accurate and faster microbiological testing in the future.

In our setting, PCR analysis for LMC pathogens was only recommended in patients with a CURB-65 score of 3–5. Therefore, we were less likely to identify LMC-pneumonias in the group prescribed antibiotics without LMC coverage and likely overestimated the proportion of LMC-pneumonias prescribed antibiotics covering LMC pneumonia. To PCR test all CAP patients or all patients prescribed antibiotics with LMC coverage [39] may lead to reduced antibiotic use, cost and length of hospital stay [40].

#### Generalizability

The relative frequency of each pathogen in our study was consistent with other studies from Denmark and Norway [41, 42]. We had a low frequency of *Streptococcus pneumoniae* compared to other European countries but a similar frequency compared to USA and Canada [4]. Concerning LMC pneumonia pathogens, this study has a low frequency compared to many other studies that reported a frequency of approximately 21% [4]. The frequency difference could be attributed to regional differences, the use of serology, and the more consistent use of PCR for LMC in other studies.

Denmark and other Scandinavian countries have a low level of LMC frequency and antibiotic resistance, and guidelines usually recommend narrow-spectrum penicillin, in contrast to non-Scandinavian countries where broad-spectrum antibiotics are often used as first-line empirical treatment [14, 16, 42–44]. Therefore these results might not apply to countries with different etiology, antibiotic resistance patterns, or guidelines for CAP. Nevertheless, the underlining observations from this study may be more broadly applicable. Inappropriate use of broad-spectrum antibiotics may be driven by diagnostic uncertainty regarding infection focus and etiology.

#### Limitations

A limitation of this study was that the population was selective and only included 8% of patients with CAP, as it was only possible to identify microbiological etiology in 13% of patients. Sputum culture and PCR are the primary microbiological tests for CAP. However, studies have shown a tendency for a low sputum culture yield of 14–17% in CAP patients because of an inability to obtain sputum samples and a low positive rate of sputum culture [45, 46]. In addition, patient factors such as age > 75 years, weakness, and ability to cough will impact the ability to produce a sputum sample. Furthermore, antibiotic treatment before sputum sampling reduces sputum culture yield, so some pathogens are particularly challenging to identify via culture [46].

Some, limitations stem from our population-based design, which relies on discharge diagnoses for pneumonia, potentially skewing results due to less robust patient selection. Furthermore, the overrepresentation of patients from Odense University Hospital, with its unique patient demographic and extensive microbiological testing, could have biased our results, specifically in antibiotic prescription patterns. The impact of these factors is challenging to quantify or account for.

## Conclusion

In conclusion, piperacillin-tazobactam was the most frequent empirical antibiotic treatment for CAP with later established clear etiology. The accuracy of empirical antibiotic treatment covering LMC pneumonia was low. We found that there was potential for improvement of empirical antibiotic treatment of CAP and that diagnostic uncertainty regarding focus and cause of infection may be major factors for unnecessary use of broad-spectrum antibiotics.

### Supplementary Information

**Additional file 1:** **Appendix 1.** List of ICD-10 codes and n included in the study. **Appendix 2.** Guidelines for microbiological testing. **Appendix 3.** Potential CAP pathogens by microbiological tests. **Appendix 4.** Extended list of empirical antibiotic treatment. **Appendix 6.** Frequency of microbiological testing in all CAP patients and frequency of positive test for a CAP pathogen.

## Data Availability

Due to Danish laws on personal data, data cannot be shared publicly. To request data, please contact the corresponding author for more information.
