# Supplementary material for: Empirical antibiotic treatment for community-acquired pneumonia and accuracy for Legionella pneumophila, Mycoplasma pneumoniae, and Clamydophila pneumoniae: a descriptive cross-sectional study of adult patients in the emergency department
Source: BMC Infect Dis. 2023 Sep 5;23:580. doi: 10.1186/s12879-023-08565-6 (PMC10481610; doi:10.1186/s12879-023-08565-6)
Supplement: Supplementary file 1 — Additional file 1: Appendix 1. List of ICD-10 codes and n included in the study. Appendix 2. Guidelines for microbiological testing. Appendix 3. Potential CAP pathogens by microbiological tests. Appendix 4. Extended list of empirical antibiotic treatment. Appendix 6. Frequency of microbiological testing in all CAP patients and frequency of positive test for a CAP pathogen. [file 12879_2023_8565_MOESM1_ESM.docx]

Appendix

Appendix 1: list of ICD-10 codes and n included in the study.

| ICD accounting for more than 0.5% | | |
| --- | --- | --- |
| ICD-10 code | Description | N (%) |
| DJ189 | Pneumonia, unspecified | 13.652 (71.4%) |
| DJ159 | Bacterial pneumonia, unspecified | 4100 (21.4%) |
| DJ158 | Other bacterial pneumonia | 327 (1.7%) |
| DJ180 | Bronchopneumonia, unspecified | 152 (0.8%) |
| DJ100 | Influenza with pneumonia, seasonal influenza virus identified | 147 (0.8%) |
| DJ111 | Influenza with other respiratory manifestations, virus not identified | 102 (0.5%) |

Table 3 the distribution of ICD-10 codes among the included CAP patients.

Appendix 2: Guidelines for microbiological testing

The treating physician was responsible for ordering microbiological diagnostics. Routine blood, urine, and sputum culture in all patients with suspected infection were recommended. Polymerase chain reaction (PCR) for Legionella pneumophila, Chlamydia pneumoniae, and Mycoplasma pneumonia was recommended in patients with a CURB-65 score of 3-5. *Legionella* urinary antigen test and *Streptococcus pneumoniae* urinary antigen test were not routine. PCR for viruses was recommended doing outbreaks, influenza season, etc.

Appendix 3: Potential CAP pathogens by microbiological tests

| Sputum culture | Blood culture |
| --- | --- |
| *Chlamydia pneumoniae*  *Haemophilus influenzae  Legionella pneumophila*  *Mycoplasma pneumoniae*  *Streptococcus pneumoniae*  *Aspergillus fumigatus*  *Hemolytic streptococci*  *Moraxella catarrhalis*  *Neisseria meningitidis*  *Staphylococcus aureus*  *Pseudomonas aeruginosa* | *Haemophilus influenzae*  *Streptococcus pneumonia*  *Hemolytic streptococci*  *group C/G*  *Pseudomonas aeruginosa* |

Potential pathogens being the cause of pneumonia based on microbiological tests.

All PCR results for LMC-pneumonias were considered etiology for pneumonia. In cases with more than one identified pathogen, the most probable cause was chosen, weighing LMC pathogens over other bacterial pathogens.

Appendix 4: extended list of empirical antibiotic treatment

| Appendix 4: Empirical antibiotic treatment < 24 hours after admission |  |
| --- | --- |
| - Piperacillin-tazobactam | 515 (32%) |
| - Benzylpenicillin monotherapy | 347 (22%) |
| - Cephalosporin | 148 (9%) |
| - Fluoroquinolone | 113 (7%) |
| - Amoxicillin/clavulanate | 96 (6%) |
| - Piperacillin-tazobactam + macrolide | 65 (4%) |
| - Benzylpenicillin + macrolide | 60 (4%) |
| - Phenoxymethylpenicillin | 41 (3%) |
| - Cephalosporin + macrolide | 41 (3%) |
| - Macrolide monotherapy | 36 (2%) |
| - Amoxicillin or ampicillin | 33 (2%) |
| - Piperacillin-tazobactam + fluoroquinolon | 28 (2%) |
| - Carbapenem | 16 (1%) |
| - Antibiotics targeting urinary tract infections | 13 (1%) |
| - Benzylpenicillin + fluoroquinolone | 10 (1%) |
| - Other | 28 (2%) |

Appendix 5: Identified viral pneumonias in all CAP patients

| Etiology | N=19133 |
| --- | --- |
| *Influenza A* | 262 (1%) |
| *Influenza B* | 295 (2%) |
| *RS virus* | 31 (<1%) |

Appendix 6: Frequency of microbiological testing in all CAP patients and frequency of positive test for a CAP pathogen

|  | Tested  N= 19133 |
| --- | --- |
| Blood culture | 14369 (75.1%) |
| Sputum culture | 7115 (37.2%) |
|  | **Positive tests** |
| Blood culture positive | 348 (1.8%) |
| Sputum culture positive | 1738 (9.1%) |
| PCR positive | 583* |

*Due lack of data on number of patients who were PCR tested, it was not possible to calculated positive percentage for PCR.
